# Investigation of Indolglyoxamide and Indolacetamide Analogues of Polyamines as Antimalarial and Antitrypanosomal Agents

**DOI:** 10.3390/md12063138

**Published:** 2014-05-28

**Authors:** Jiayi Wang, Marcel Kaiser, Brent R. Copp

**Affiliations:** 1 School of Chemical Sciences, University of Auckland, Private Bag 92019, Auckland 1142, New Zealand; E-Mail: jliu156@aucklanduni.ac.nz; 2 Swiss Tropical and Public Health Institute, Socinstrasse 57, PO Box, Basel CH-4002, Switzerland; E-Mail: marcel.kaiser@unibas.ch; 3 University of Basel, Basel CH-4003, Switzerland

**Keywords:** marine natural products, protozoa, malaria, *Plasmodium falciparum*, *Trypanosoma brucei rhodesiense*, polyamine, indolglyoxamide, alkaloid

## Abstract

Pure compound screening has previously identified the indolglyoxylamidospermidine ascidian metabolites didemnidine A and B (**2** and **3**) to be weak growth inhibitors of *Trypanosoma brucei rhodesiense* (IC_50_ 59 and 44 μM, respectively) and *Plasmodium falciparum* (K1 dual drug resistant strain) (IC_50_ 41 and 15 μM, respectively), but lacking in selectivity (L6 rat myoblast, IC_50_ 24 μM and 25 μM, respectively). To expand the structure–activity relationship of this compound class towards both parasites, we have prepared and biologically tested a library of analogues that includes indoleglyoxyl and indoleacetic “capping acids”, and polyamines including spermine (PA3-4-3) and extended analogues PA3-8-3 and PA3-12-3. 7-Methoxy substituted indoleglyoxylamides were typically found to exhibit the most potent antimalarial activity (IC_50_ 10–92 nM) but with varying degrees of selectivity *versus* the L6 rat myoblast cell line. A 6-methoxyindolglyoxylamide analogue was the most potent growth inhibitor of *T.* *brucei* (IC_50_ 0.18 μM) identified in the study: it, however, also exhibited poor selectivity (L6 IC_50_ 6.0 μM). There was no apparent correlation between antimalarial and anti-*T. brucei* activity in the series. *In vivo* evaluation of one analogue against *Plasmodium berghei* was undertaken, demonstrating a modest 20.9% reduction in parasitaemia.

## 1. Introduction

Alkyl amines belonging to the polyamine family [1] are widely distributed in nature, being isolated from a diverse range of terrestrial and marine sources. From the simple diamines putresine and cadaverine through to more complex examples of spermidine and spermine, polyamines have been reported to exhibit biological activities towards a large number of cellular targets and processes. While *N*-alkyl derivatives are generally cytotoxic or act synergistically with cytotoxins [2,3,4], examples have been reported to act as potent epigenetic modulators [5,6,7], to act as antioxidants [8], and to exhibit anti-trypanosomal [9,10] and anti-malarial properties [11,12,13,14,15,16].

**Figure 1:**
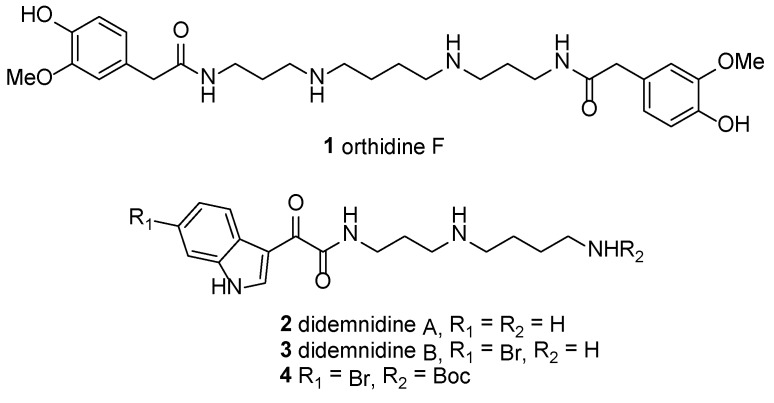
Structures of orthidine F (**1**); didemnidine A (**2**) and B (**3**) and analogue **4**.

As part of our own continuing search for new natural product leads for the development of treatments for neglected human diseases [17,18,19,20,21], we recently reported the discovery of polyamine alkaloids orthidine F (**1**) [22,23] and didemnidines A (**2**) and B (**3**) [24] as *in vitro* growth inhibitors of *Plasmodium falciparum* (K1 dual drug-resistant strain) (Figure 1). In the case of orthidine F, the antimalarial potency of the natural product (IC_50_ 0.89 μM) [23] was increased substantially (IC_50_ 1.3 nM) by undertaking a structure–activity relationship study [25], which also identified optimal structural attributes for antimalarial activity to be either a polyamine PA3-8-3 or PA3-12-3 [1] scaffold, and bearing 1, ω-disubstitution. Didemnidines A and B were found to be more modest growth inhibitors of both *P. falciparum* (IC_50_ 41 and 15 μM, respectively) and *Trypanosoma brucei rhodesiense* (IC_50_ 59 and 44 μM, respectively) [24]. Analogue **4**, prepared during the synthesis of **3**, was identified as the most active anti-protozoal compound in the limited series (*Pf* IC_50_ 8.4 μM, *Tbr* IC_50_ 9.9 μM), again suggesting that 1, ω-disubstitution of this alkaloid family might lead to the identification of more active examples.

Herein we report the results of a structure–activity relationship study investigating the influence of indole substitution, the requirement for the side chain keto group and nature of the polyamine core to the observed anti-protozoal activity of didemnidines A and B. The library was evaluated for antimalarial activity against the NF54 drug sensitive strain of *P. falciparum*, for anti-trypanosomal activity against *Trypanosoma brucei rhodesiense* and for cytotoxicity towards the non-malignant L6 rat myoblast cell line. One analogue was also tested for *in vivo* antimalarial activity against *Plasmodium berghei* in mice.

## 2. Results and Discussion

### 2.1. Chemistry

**Figure 2:**
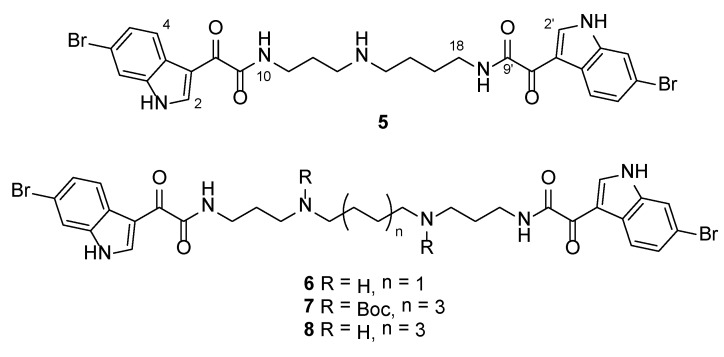
Structures of 6-bromoindolglyoxylamide analogues **5**–**8**.

Reaction of each of spermidine, spermine and di-*tert*-butyl octane-1,8-diylbis ((3-aminopropyl)carbamate) [25] with 2-(6-bromoindol-3-yl)glyoxylic acid [24] using PyBop as the coupling agent afforded, after chromatographic purification, analogues **5**–**7** in yields of 58%, 86% and 26%, respectively (Figure 2). Subsequent removal of the Boc groups present in **7** with TFA in CH_2_Cl_2_ gave tetraaminediamide **8** as the TFA salt.

**Figure 3:**
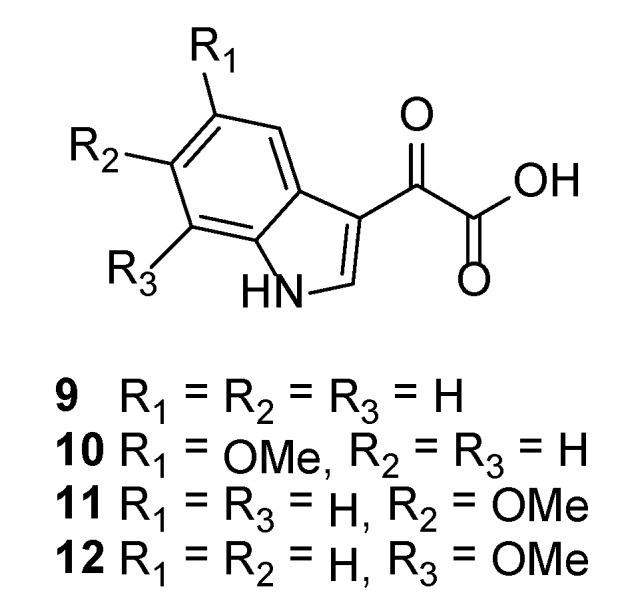
Structures of indolyl-2-oxoacetic acids **9**–**12**.

Previous studies by us have correlated electron-rich aryl substituents with enhanced anti-protozoal activity for 1, ω-disubstituted polyamines [23,25]. To explore similar properties in the context of the didemnidines, we prepared 2-(1*H*-indol-3-yl)-2-oxoacetic acid (**9**) and the 5-, 6- and 7-methoxy analogues (**10**–**12**) (Figure 3) via a literature method [26].

**Figure 4:**
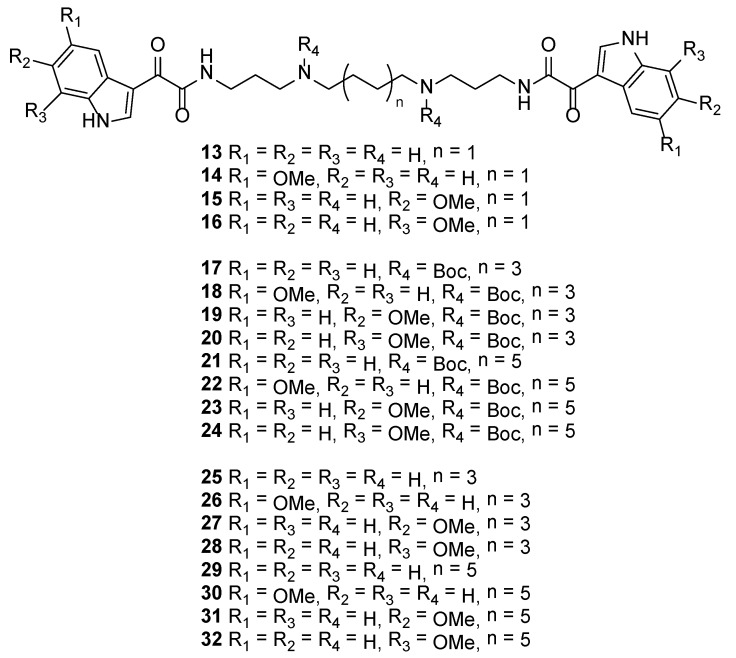
Structures of indolglyoxylamide analogues **13**–**32**.

Using each of **9**–**12**, PyBop-mediated coupling with spermine, di-*tert*-butyl octane-1,8-diylbis((3-aminopropyl)carbamate) [25] and di-*tert*-butyl dodecane-1,12-diylbis((3-aminopropyl)carbamate) [27,28], afforded analogues **13**–**24**, while Boc group deprotection, again with TFA in CH_2_Cl_2_, gave tetraamine diamides **25**–**32** as their corresponding di-TFA salts (Figure 4).

**Figure 5:**
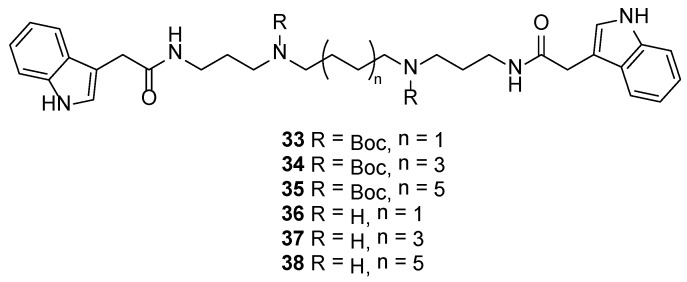
Structures of indolacetamide analogues **33**–**38**.

We finally sought to explore the influence of the sidechain keto group on the observed activity of the didemnidines. Thus PyBOP or HATU-mediated coupling of commercially available indole-3-acetic acid with di-*tert*-butyl butane-1,4-diylbis((3-aminopropyl)carbamate) [25,27], di-*tert*-butyl octane-1,8-diylbis((3-aminopropyl)carbamate) [25] and di-*tert*-butyl dodecane-1,12-diylbis ((3-aminopropyl)carbamate) [27,28] afforded polyamine amides **33**–**35** with yields of 39%, 35% and 44%, respectively (Figure 5). Subsequent removal of the Boc groups with TFA in CH_2_Cl_2_ gave tetraamine diamides **36**–**38** as TFA salts.

### 2.2. Biological Activities

#### 2.2.1. *In Vitro* Biological Evaluation

The library of target analogues were screened against the protozoa *T. brucei rhodesiense* and *P. falciparum* and for cytotoxicity towards the rat skeletal myoblast cell line L6 and the results are summarized in Table 1.

**Table 1:** Anti-trypanosomal, antimalarial and cytotoxic activities of **2**–**8**, **13**–**16**, **18**–**38**.

Entry	Compound	IC_50_ (μM ^a^)			Pf SI ^e^
		*T. b. rhod.* ^b^	*P. falc* *.* ^c^	L6 ^d^	
1	**2 ^f^**	59	41 ^g^	24	0.59
2	**3 ^f^**	44	15 ^g^	25	1.7
3	**4 ^f^**	9.9	8.4 ^g^	25	3.0
4	**5**	NT ^h^	0.25	5.5	22
5	**6**	NT	0.36	7.7	21
6	**7**	NT	0.27	92	340
7	**8**	NT	0.41	5.6	14
8	**13**	NT	0.12	60	500
9	**14**	NT	0.47	56	120
10	**15**	NT	0.50	54	110
11	**16**	45	1.3	62	48
12	**18 **	6.2	0.13	≥120	≥920
13	**19**	6.1	0.14	≥120	≥920
14	**20**	61	0.092	≥120	≥1300
15	**21**	61	1.8	≥120	≥67
16	**22**	5.2	0.36	19	53
17	**23**	62	1.9	≥110	≥58
18	**24**	63	1.7	≥110	≥65
19	**25**	2.5	0.11	19	170
20	**26**	0.78	0.13	13	100
21	**27**	2.2	0.17	21	120
22	**28**	2.1	0.12	6.6	55
23	**29**	0.27	0.033	2.3	70
24	**30**	0.27	0.20	17	85
25	**31**	0.18	0.24	6.0	25
26	**32**	0.26	0.010	2.1	210
27	**33**	7.1	0.16	19	120
28	**34**	NT	0.30	5.0	17
29	**35**	NT	0.80	45	56
30	**36**	75	0.18	74	410
31	**37**	NT	0.15	64	430
32	**38**	NT	0.12	19	160
	Melarsoprol ^i^	0.005			
	Chloroquine ^i^		0.004		
	Podophyllotoxin ^i^			0.019	

^a^ IC_50_ values reported are the average of two independent assays. Assay protocols are described in [29]; ^b^ *Trypanosoma brucei rhodesiense*, STIB 900 strain, trypomastigotes stage; ^c^ *Plasmodium falciparum*, NF54 strain, IEF stage; ^d^ L6 rat skeletal myoblast cell line; ^e^ Selectivity index for *P. falciparum* = IC_50_ L6/IC_50_ *Pf*; ^f^ Data taken from reference [24]; ^g^ *Plasmodium falciparum*, K1 strain, IEF stage; ^h^ not tested; ^i^ Melarsoprol, chloroquine and podophyllotoxin were used as positive controls.

Bromoindoles **5**–**8** (entries 4–7) were all more active against *Pf* than the original natural products **2** and **3** and analogue **4**. Only one analogue however, *bis*-*tert*-carboxylcarbonyl protected **7**, demonstrated some degree of selectivity with L6 cytotoxicity of IC_50_ 92 μM and a selectivity index of 340 (entry 6). Of spermine analogues **13**–**16** (entries 8–11), debromoindole **13** (entry 8) exhibited good potency towards *Pf* (IC_50_ 0.12 μM) with improved selectivity (L6 IC_50_ 60 μM, Pf SI 500). All of the *tert*-butoxycarbonyl protected PA3-8-3 analogues tested (**18**–**20**, entries 12–14) exhibited acceptable levels of selectivity, with 7-methoxyindole **20** (entry 14) identified as being a potent growth inhibitor of *Pf* (IC_50_ 92 nM) with excellent selectivity (L6 IC_50_ ≥ 120 μM, Pf SI ≥ 1300). The corresponding Boc-protected PA3-12-3 analogues **21**–**24** (entries 15–18) were less active towards *Pf* and only modestly selective. Removal of the Boc group afforded **25**–**32** (entries 19–26), of which PA3-12-3 analogues **29** (entry 23) and **32** (entry 26) were identified as potent anti-*Pf* compounds but with only moderate selectivity (*Pf* SI 70 and 210, respectively). Using the rather crude tool of averaging anti-*Pf* IC_50_ values for all PA3-8-3 and PA3-12-3 analogues indicates that those that contain the PA3-8-3 core are typically 6–7 times more active (average IC_50_ 0.13 μM) than the corresponding PA3-12-3 analogues (average IC_50_ 0.89 μM). Examination of the anti-*Pf* data observed for the set of indole-3-acetic acid analogues **33**–**38** (entries 27–32) suggested little influence of the keto group in the sidechain for potency, but that the analogues were typically of similar or more potent cytotoxicity. Compared to our previous studies of antimalarial benzamide, phenylacetamide, phenethylamide and phenyl-3-propanamide polyamine analogues [23,25], the present results indicate indoleglyoxyl and indoleacetamides to be more cytotoxic and less potent against *Pf*, suggesting future studies should be directed towards the former classes of “capping acids”.

In the case of anti-*Trypanosoma brucei* *rhodesiense* activity, PA3-12-3 analogues **29**–**32** (entries 23–26) were the most active (IC_50_ 0.18–0.27 μM), but unfortunately were also some of the more cytotoxic diamides prepared.

#### 2.2.2. *In Vivo* Anti-Malarial Evaluation

Analogue **20** was selected for *in vivo* evaluation in *Plasmodium berghei* infected mice. Using a standard test protocol [30], a repeated ip dose of 50 (mg/kg)/day for four days led to a 20.9% reduction in parasitaemia. No increase in mean survival time was observed. 

## 3. Experimental Section

### 3.1. General

HRMS data were acquired on a Bruker micrOTOF-QII mass spectrometer (Bruker Daltonik GmbH, Bremen, Germany). Infrared spectra were recorded on a Perkin-Elmer Spectrum 100 Fourier-transform IR spectrometer (Perkin Elmer, Waltham, MA,) equipped with a universal ATR accessory. Melting points were obtained on an Electrothermal melting point apparatus and are uncorrected. NMR spectra were recorded using either a Bruker Avance DRX 300 or 400 spectrometer (Bruker BioSpin GmbH, Rheinstetten, Germany) operating at 300 MHz or 400 MHz for ^1^H nuclei and 75 MHz or 100 MHz for ^13^C nuclei. Resonance assignments were made by interpretation of 2D data. NMR assignments marked by a superscripted letter are interchangeable. Proto-deutero solvent signals were used as internal references (DMSO-*d*_6_: δ_H_ 2.50, δ_C_ 39.52; CDCl_3_: δ_H_ 7.25, δ_C_ 77.0; CD_3_OD: δ_H_ 3.30, δ_C_ 49.05). Flash column chromatography was performed using reversed-phase Merck Lichroprep RP-18 (Merck, Manakau, New Zealand), or Kieselgel 60 PF silica gel (Merck, Manakau, New Zealand). Thin layer chromatography used 0.2 mm thick plates of Kiesegel F_254_ (Merck, Manakau, New Zealand). The syntheses of 2-(1*H*-indol-3-yl)-2-oxoacetic acid (**9**) [26], 2-(6-bromo-1*H*-indol-3-yl)-2-oxoacetic acid [24], di-*tert*-butyl butane-1,4-diylbis((3-aminopropyl)carbamate) [25,27], di-*tert*-butyl octane-1,8-diylbis((3-aminopropyl)carbamate) [25] and di-*tert*-butyl dodecane-1,12-diylbis((3-aminopropyl)carbamate) [27,28] have been reported previously.

### 3.2. Synthetic Procedures

#### 3.2.1. General Procedure A: Amide Bond Formation

To a solution of carboxylic acid (2.05 equiv.), diamine (1 equiv.), and PyBOP (2.05 equiv.) in DMF (1 mL) was added Et_3_N (3 equiv.). The reaction mixture was allowed to stir under N_2_ at room temperature for 23 h. The solution was dried in vacuo and the crude reaction product purified by C_8_ reversed-phase column chromatography (20%–30% MeOH/H_2_O (+0.05%TFA)) to afford the target diamide as the bis-trifluoroacetate salt or by silica gel column chromatography (0%–1% MeOH in CH_2_Cl_2_) to afford the target diamide as the free base.

#### 3.2.2. General Procedure B: Removal of Boc Protecting Group

A solution of *tert*-butyl-carbamate derivative in CH_2_Cl_2_ (2 mL) and TFA (0.2 mL) was stirred at room temperature under N_2_ for 2 h, then dried *in vacuo* to afford the deprotected analogue. In some cases the product required no further purification, while in other cases, purification was achieved by C_18_ reversed-phase column chromatography eluting with 0%–50% MeOH/H_2_O (+0.05% TFA).

#### 3.2.3. 4-(2-(6-Bromo-1*H*-indol-3-yl)-2-oxoacetamido)-*N*-(3-(2-(6-bromo-1*H*-indol-3-yl)-2-oxoacetamido)propyl)butan-1-aminium 2,2,2-trifluoroacetate (**5**)

Using general procedure A, 2-(6-bromo-1*H*-indol-3-yl)-2-oxoacetic acid [24] (60 mg, 0.21 mmol), spermidine (15 mg, 0.10 mmol), PyBOP (109 mg, 0.21 mmol) and Et_3_N (83 μL, 0.60 mmol) afforded **5** as a yellow gum (37 mg, 58% yield).

R*_f_* = 0.26 (CH_2_Cl_2_:MeOH:TEA 4:1:0.01); IR ν_max_ (ATR) 3247, 1658, 1602, 1503, 1135, 841 cm^−1^; ^1^H NMR (DMSO-*d*_6_, 400 MHz) δ_H_ 12.38 (2H, br s, NH-1 and NH-1′), 8.91 (1H, t, *J* = 6.0 Hz, NH-10), 8.80 (1H, t, *J* = 6.1 Hz, NH-19), 8.78 (2H, d, *J* = 4.1 Hz, H-2 and H-2′), 8.42 (2H, br s, NH_2_-14), 8.15 (2H, d, *J* = 8.5 Hz, H-4 and H-4′), 7.75 (2H, d, *J* = 1.5 Hz, H-7 and H-7′), 7.40 (2H, dd, *J* = 8.5, 1.5 Hz, H-5 and H-5′), 3.30 (2H, td, *J* = 7.2, 6.0 Hz, H_2_-11), 3.25 (2H, td, *J* = 6.1, 5.8 Hz, H_2_-18), 3.02–2.88 (4H, m, H_2_-13 and H_2_-15), 1.85 (2H, tt, *J* = 7.2, 7.2 Hz, H_2_-12), 1.67–1.53 (4H, m, H_2_-16 and H_2_-17); ^13^C NMR (DMSO-*d*_6_, 100 MHz) δ_C_ 182.2 (C-8^a^), 181.8 (C-8′^a^), 163.5 (C-9), 163.4 (C-9′), 139.3 (C-2^b^), 139.3 (C-2′^b^), 137.3 (C-7a and C-7a′), 125.5 (C-5 and C-5′), 125.4 (C-3a^c^), 125.3 (C-3a′^c^), 122.9 (C-4 and C-4′), 116.0 (C-6^d^), 116.0 (C-6′^d^), 115.4 (C-7 and C-7′), 112.1 (C-3^e^), 112.1 (C-3′^e^), 46.6 (C-15), 44.8 (C-13), 37.9 (C-18), 35.8 (C-11), 25.9 (C-16^f^), 25.7 (C-12), 23.2 (C-17^f^); (+)-HRESIMS *m/z* 644.0506 [M + H]^+^ (calcd for C_27_H_28_^79^Br_2_N_5_O_4_, 644.0503).

#### 3.2.4. *N*^1^,*N*^4^-Bis(3-(2-(6-bromo-1*H*-indol-3-yl)-2-oxoacetamido)propyl)butane-1,4-diaminium 2,2,2-trifluoroacetate (**6**)

Using general procedure A, 2-(6-bromo-1*H*-indol-3-yl)-2-oxoacetic acid [24] (50 mg, 0.18 mmol), spermine (17 mg, 0.083 mmol), PyBOP (91 mg, 0.18 mmol) and Et_3_N (69 μL, 0.50 mmol) afforded **6** as a brown oil (50 mg, 86% yield).

R*_f_* = 0.17 (CH_2_Cl_2_:MeOH:TEA 1:1:0.01); IR ν_max_ (ATR) 3278, 1672, 1628, 1441, 1201, 1131, 799, 721, 686 cm^−1^; ^1^H NMR (DMSO-*d*_6_, 400 MHz) δ_H_ 12.41 (1H, br s, NH-1), 8.91 (1H, t, *J* = 6.3 Hz, NH-10), 8.78 (1H, d, *J* = 3.5 Hz, H-2), 8.58 (2H, br s, NH_2_-14), 8.15 (1H, d, *J* = 8.5 Hz, H-4), 7.76 (1H, d, *J* = 1.8 Hz, H-7), 7.41 (1H, dd, *J* = 8.5, 1.8 Hz, H-5), 3.30 (2H, td, *J* = 6.9, 6.3 Hz, H_2_-11), 2.99–2.89 (4H, m, H_2_-13 and H_2_-15), 1.85 (2H, tt, *J* = 6.9, 6.9 Hz, H_2_-12), 1.68–1.56 (2H, m, H_2_-16); ^13^C NMR (DMSO-*d*_6_, 100 MHz) δ_C_ 181.8 (C-8), 163.5 (C-9), 139.3 (C-2), 137.2 (C-7a), 125.5 (C-5), 125.3 (C-3a), 122.9 (C-4), 116.0 (C-6), 115.4 (C-7), 112.1 (C-3), 46.1 (C-15), 44.7 (C-13), 35.9 (C-11), 25.7 (C-12), 22.7 (C-16); (+)-HRESIMS *m/z* 701.1087 [M + H]^+^ (calcd for C_30_H_35_^79^Br_2_N_6_O_4_, 701.1081).

#### 3.2.5. Di-*tert*-butyl Octane-1,8-diylbis((3-(2-(6-bromo-1*H*-indol-3-yl)-2-oxoacetamido)propyl)carbamate) (**7**)

Using general procedure A, 2-(6-bromo-1*H*-indol-3-yl)-2-oxoacetic acid [24] (0.12 g, 0.42 mmol), di-*tert*-butyl octane-1,8-diylbis((3-aminopropyl)carbamate) [25] (91 mg, 0.20 mmol), PyBOP (0.22 g, 0.42 mmol) and Et_3_N (83 μL, 0.60 mmol) afforded **7** as a peach gum (51 mg, 26% yield).

R*_f_* = 0.60 (hexane:EtOAc 3:7); IR ν_max_ (ATR) 3226, 2929, 1666, 1631, 1417, 1156, 793, 633 cm^−1^; ^1^H NMR (DMSO-*d*_6_, 400 MHz) δ_H_ 12.27 (1H, br s, NH-1), 8.78 (1H, s, H-2), 8.74 (1H, br s, NH-10), 8.15 (1H, d, *J* = 8.4 Hz, H-4), 7.73 (1H, d, *J* = 1.7 Hz, H-7), 7.39 (1H, dd, *J* = 8.4, 1.7 Hz, H-5), 3.18 (2H, td, *J* = 7.1, 6.9 Hz, H_2_-11), 3.13 (2H, t, *J* = 7.1 Hz, H_2_-13), 3.08 (2H, t, *J* = 7.2 Hz, H_2_-15), 1.75–1.64 (2H, m, H_2_-12), 1.46–1.32 (2H, m, H_2_-16), 1.36 (9H, s, 3H_3_-21), 1.26–1.11 (4H, m, H_2_-17 and H_2_-18); ^13^C NMR (DMSO-*d*_6_, 100 MHz) δ_C_ 182.1 (C-8), 163.2 (C-9), 154.7 (C-19), 139.2 (C-2), 137.2 (C-7a), 125.4 (C-5), 125.3 (C-3a), 122.9 (C-4), 115.9 (C-6), 115.3 (C-7), 112.1 (C-3), 78.2 (C-20), 46.3 (C-15), 44.4, 44.0 (C-13), 36.4 (C-11), 28.7 (C-18), 28.0 (C-21), 27.7 (C-16 and C-12), 26.1 (C-17); (+)-HRESIMS *m/z* 979.2573 [M + Na]^+^ (calcd for C_44_H_58_^79^Br_2_N_6_NaO_8_, 979.2575).

#### 3.2.6. *N*^1^,*N*^8^-Bis(3-(2-(6-bromo-1*H*-indol-3-yl)-2-oxoacetamido)propyl)octane-1,8-diaminium 2,2,2-trifluoroacetate (**8**)

Using general procedure B, reaction of **7** (9 mg, 9.4 μmol) in CH_2_Cl_2_ (1.7 mL) with TFA (0.3 mL) afforded **8** as a yellow gum (9 mg, quant. yield) which required no further purification.

R*_f_* = 0.19 (CH_2_Cl_2_:MeOH:TEA 4:1:0.01); IR ν_max_ (ATR) 3321, 3180, 1717, 1597, 1184, 1133, 719, 655 cm^−1^; ^1^H NMR (DMSO-*d*_6_, 400 MHz) δ_H_ 12.46 (1H, br s, NH-1), 8.91 (1H, t, *J* = 6.3 Hz, NH-10), 8.77 (1H, s, H-2), 8.68 (2H, br s, NH_2_-14), 8.15 (1H, d, *J* = 8.4, H-4), 7.75 (1H, d, *J* = 1.9 Hz, H-7), 7.40 (1H, dd, *J* = 8.4, 1.9 Hz, H-5), 3.29 (2H, td, *J* = 7.3, 6.3 Hz, H_2_-11), 2.95–2.88 (2H, m, H_2_-13), 2.88–2.82 (2H, m, H_2_-15), 1.86 (2H, tt, *J* =7.3, 6.6 Hz, H_2_-12), 1.63–1.52 (2H, m, H_2_-16), 1.34–1.21 (4H, m, H_2_-17 and H_2_-18); ^13^C NMR (DMSO-*d*_6_, 100 MHz) δ_C_ 181.9 (C-8), 163.5 (C-9), 139.2 (C-2), 137.2 (C-7a), 125.4 (C-5), 125.3 (C-3a), 122.9 (C-4), 116.0 (C-6), 115.4 (C-7), 112.0 (C-3), 46.7 (C-15), 44.6 (C-13), 35.9 (C-11), 28.3 (C-18), 25.8 (C-12^a^), 25.6 (C-17^a^), 25.4 (C-16^a^); (+)-HRESIMS *m/z* 757.1708 [M + H]^+^ (calcd for C_34_H_43_^79^Br_2_N_6_O_4_, 757.1707).

#### 3.2.7. 2-(5-Methoxy-1*H*-indol-3-yl)-2-oxoacetic Acid (**10**)

The target compound **10** was prepared using a previously published method [26]. To a solution of 5-methoxyindole (0.15 g, 0.985 mmol) in anhydrous diethyl ether (18 mL) was added oxalyl chloride (0.13 mL, 1.48 mmol) dropwise at 0 °C. Reaction was stirred at 0 °C for 2 h, during which time an orange precipitate was formed. Saturated aq. NaHCO_3_ (6 mL) was added, and the reaction mixture heated at reflux for 2 h. After cooling to r.t., 10% HCl was added to adjust the solution to pH 1, the resulting precipitate filtered and dried under vacuum to yield **10** as an orange powder (0.20 g, 91% yield).

Mp 236 °C decomp. (lit. [31] 248 °C); R*_f_* = 0.09 (20% MeOH/EtOAc); IR ν_max_ (ATR) 3157, 2918, 1732, 1612, 1475, 1460, 1420, 1438, 1273, 1196, 1166, 913, 818, 809, 760, 709 cm^−1^; ^1^H NMR (DMSO-*d*_6_, 400 MHz) δ_H_ 12.29 (1H, br s, NH), 8.32 (1H, d, *J* = 3.4 Hz, H-2), 7.67 (1H, d, *J* = 2.5 Hz, H-4), 7.44 (1H, d, *J* = 8.8 Hz, H-7), 6.91 (1H, dd, *J* = 8.8, 2.5 Hz, H-6), 3.79 (3H, s, H_3_-10), OH not observed; ^13^C NMR (DMSO-*d*_6_, 100 MHz) δ_C_ 180.8 (C-8), 165.5 (C-9), 156.2 (C-5), 138.0 (C-2), 131.5 (C-7a), 126.6 (C-3a), 113.6 (C-7), 113.4 (C-6), 112.3 (C-3), 103.2 (C-4), 55.5 (C-10); (−)-HRESIMS *m/z* 218.0470 [M − H]^−^ (calcd for C_11_H_8_NO_4_, 218.0459).

#### 3.2.8. 2-(6-Methoxy-1*H*-indol-3-yl)-2-oxoacetic Acid (**11**)

The target compound **11** was prepared using a previously published method [26]. To a solution of 6-methoxyindole (0.13 g, 0.866 mmol) in anhydrous diethyl ether (10 mL) was added oxalyl chloride (0.11 mL, 1.30 mmol) dropwise at 0 °C. The reaction mixture was allowed to stir at 0 °C for 3 h before it was warmed to r.t. Saturated aq. NaHCO_3_ (10 mL) was then added, and the reaction mixture heated at reflux for 1 h. After cooling to r.t., the pH of the reaction mixture was adjusted to 1 using 10% HCl. The resulting green precipitate was filtered, washed with cold diethyl ether (30 mL) and dried under vacuum to yield **11** as a green powder (0.18 g, 97% yield) which was used in the next step without further purification.

Mp 226 °C decomp.; R*_f_* = 0.09 (20% MeOH/EtOAc); IR ν_max_ (ATR) 3167, 1733, 1608, 1394, 1142, 1093, 710, 653 cm^−1^; ^1^H NMR (DMSO-*d*_6_, 400 MHz) δ_H_ 12.11 (1H, br s, NH), 8.30 (1H, s, H-2), 8.03 (1H, d, *J* = 8.6 Hz, H-4), 7.03 (1H, s, H-7), 6.90 (1H, dd, *J* = 8.6, 1.8 Hz, H-5), 3.80 (3H, s, H_3_-10), OH not observed; ^13^C NMR (DMSO-*d*_6_, 100 MHz) δ_C_ 180.8 (C-8), 165.3 (C-9), 156.9 (C-6), 137.7 (C-7a), 137.2 (C-2), 121.8 (C-4), 119.4 (C-3a), 112.5 (C-3), 112.3 (C-5), 95.8 (C-7), 55.3 (C-10); (+)-HRESIMS *m/z* 220.0615 [M + H]^+^ (calcd for C_11_H_10_NO_4_, 220.0604).

#### 3.2.9. 2-(7-Methoxy-1*H*-indol-3-yl)-2-oxoacetic Acid (**12**)

The target compound **12** was prepared using a previously published method [26]. To a solution of 7-methoxyindole (0.30 g, 2.04 mmol) in anhydrous diethyl ether (9 mL) was added oxalyl chloride (0.52 mL, 6.11 mmol) dropwise at 0 °C. The reaction mixture was stirred at 0 °C for 1.5 h, followed by dropwise addition of saturated aq. NaHCO_3_ (10 mL), and then heated at reflux for 20.5 h. After cooling to r.t., 10% HCl was added to the reaction mixture to adjust pH to 1 and the resulting brown precipitate was filtered, washed with cold diethyl ether (20 mL), and dried under vacuum to yield **12** as a brown solid (0.45 g, quant. yield) which was used in the next step without further purification.

Mp 206 °C decomp.; R*_f_* = 0.14 (20% MeOH/EtOAc); IR ν_max_ (ATR) 3129, 1712, 1615, 1567, 1450, 1234, 1221, 956, 782 cm^−1^; ^1^H NMR (DMSO-*d*_6_, 300 MHz) δ_H_ 12.51 (1H, br s, NH), 8.23 (1H, d, *J* = 2.9 Hz, H-2), 7.74 (1H, d, *J* = 7.9 Hz, H-4), 7.19 (1H, t, *J* = 7.9 Hz, H-5), 6.87 (1H, d, *J* = 7.9 Hz, H-6), 3.95 (3H, s, H_3_-10), OH not observed; ^13^C NMR (DMSO-*d*_6_, 100 MHz) δ_C_ 180.8 (C-8), 165.2 (C-9), 146.5 (C-7), 136.8 (C-2), 127.2 (C-3a), 126.6 (C-7a), 123.7 (C-5), 113.6 (C-4), 112.9 (C-3), 104.6 (C-6), 55.4 (C-10); (−)-HRESIMS *m/z* 220.0603 [M + H]^+^ (calcd for C_11_H_8_NO_4_, 220.0604).

#### 3.2.10. *N*^1^,*N*^4^-Bis(3-(2-(1*H*-indol-3-yl)-2-oxoacetamido)propyl)butane-1,4-diaminium 2,2,2-trifluoroacetate (**13**)

Using general procedure A, 2-(1*H*-indol-3-yl)-2-oxoacetic acid (**9**) (100 mg, 0.53 mmol), spermine (49 mg, 0.24 mmol), PyBOP (275 mg, 0.53 mmol) and Et_3_N (107 μL, 1.4 mmol) afforded **13** as a creamy gum (191 mg, quant. yield).

R*_f_* = 0.26 (CH_2_Cl_2_:MeOH:TEA 4:1:0.01); IR ν_max_ (ATR) 3361, 3093, 1679, 1626, 1428, 1125, 721 cm^−1^; ^1^H NMR (DMSO-*d*_6_, 400 MHz) δ_H_ 12.29 (1H, s, NH-1), 8.89 (1H, t, *J* = 6.0 Hz, NH-10), 8.76 (1H, s, H-2), 8.26–8.20 (1H, m, H-4), 7.57–7.51 (1H, m, H-7), 7.31–7.22 (2H, m, H-5 and H-6), 3.33–3.26 (2H, m, H_2_-11), 2.99–2.89 (4H, m, H_2_-13 and H_2_-15), 1.92–1.79 (2H, m, H_2_-12), 1.67–1.58 (2H, m, H_2_-16); ^13^C NMR (DMSO-*d*_6_, 100 MHz) δ_C_ 181.7 (C-8), 163.8 (C-9), 138.5 (C-2), 136.3 (C-7a), 126.2 (C-3a), 123.5 (C-5^a^), 122.6 (C-6^a^), 121.2 (C-4), 112.6 (C-7), 112.1 (C-3), 46.1 (C-15^b^), 44.8 (C-13^b^), 35.8 (C-11), 25.7 (C-12), 22.8 (C-16); (+)-HRESIMS *m/z* 545.2866 [M + H]^+^ (calcd for C_30_H_37_N_6_O_4_, 545.2871).

#### 3.2.11. *N*^1^,*N*^4^-Bis(3-(2-(5-methoxy-1*H*-indol-3-yl)acetamido)propyl)butane-1,4-diaminium 2,2,2-trifluoroacetate (**14**)

Using general procedure A, 2-(5-methoxy-1*H*-indol-3-yl)-2-oxoacetic acid (**10**) (60 mg, 0.27 mmol), spermine (25 mg, 0.12 mmol), PyBOP (142 mg, 0.27 mmol), and Et_3_N (103 μL, 0.74 mmol) afforded **14** as a green gum (19 mg, 26% yield).

R*_f_* = 0.06 (MeOH:TEA 5:0.01); IR ν_max_ (ATR) 3347, 1679, 1438, 1127, 721 cm^−1^; ^1^H NMR (DMSO-*d*_6_, 400 MHz) δ_H_ 12.21 (1H, br s, NH-1), 8.84 (1H, t, *J* = 5.8 Hz, NH-10), 8.68 (1H, s, H-2), 7.74 (1H, d, *J* = 1.8 Hz, H-4), 7.44 (1H, d, *J* = 8.6 Hz, H-7), 6.90 (1H, dd, *J* = 8.6, 1.8 Hz, H-6), 3.79 (3H, s, H_3_-17), 3.33–3.25 (2H, td, *J* = 6.8, 5.8 Hz, H_2_-11), 2.91–2.80 (4H, m, H_2_-13 and H_2_-15), 1.88–1.77 (2H, m, H_2_-12), 1.65–1.58 (2H, m, H_2_-16); ^13^C NMR (DMSO-*d*_6_, 100 MHz) δ_C_ 181.7 (C-8), 163.9 (C-9), 156.1 (C-5), 138.5 (C-2), 131.1 (C-7a), 127.2 (C-3a), 113.4 (C-7), 112.9 (C-6), 112.0 (C-3), 103.5 (C-4), 55.3 (C-17), 46.7 (C-15), 45.0 (C-13), 36.1 (C-11), 26.3 (C-12), 23.8 (C-16); (+)-HRESIMS *m/z* 605.3089 [M + H]^+^ (calcd for C_32_H_41_N_6_O_6_, 605.3082).

#### 3.2.12. *N*^1^,*N*^4^-Bis(3-(2-(6-methoxy-1*H*-indol-3-yl)acetamido)propyl)butane-1,4-diaminium 2,2,2-trifluoroacetate (**15**)

Using general procedure A, 2-(6-methoxy-1*H*-indol-3-yl)-2-oxoacetic acid (**11**) (70 mg, 0.32 mmol), spermine (29 mg, 0.15 mmol), PyBOP (116 mg, 0.32 mmol) and Et_3_N (121 μL, 0.87 mmol) afforded **15** as a yellow solid (44 mg, 52% yield).

Mp 223 °C decomp.; R*_f_* = 0.03 (MeOH:TEA 5:0.01); IR ν_max_ (ATR) 3173, 2780, 1655, 1600, 1435, 1162, 665 cm^−1^; ^1^H NMR (DMSO-*d*_6_, 400 MHz) δ_H_12.07 (1H, s, NH-1), 8.86 (1H, t, *J* = 6.3Hz, NH-10), 8.64 (1H, d, *J* = 3.1 Hz, H-2), 8.50 (1H, br s, NH-14), 8.07 (1H, d, *J* = 8.7 Hz, H-4), 7.04 (1H, d, *J* = 2.4 Hz, H-7), 6.89 (1H, dd, *J* = 8.7, 2.4 Hz, H-6), 3.79 (3H, s, H_3_-17), 3.29 (2H, td, *J* = 7.2, 6.3 Hz, H_2_-11), 3.02–2.86 (4H, m, H_2_-13 and H_2_-15), 1.92–1.80 (2H, m, H_2_-12), 1.68–1.58 (2H, m, H_2_-16); ^13^C NMR (DMSO-*d*_6_, 100 MHz) δ_C_ 181.6 (C-8), 163.8 (C-9), 156.8 (C-6), 137.7 (C-2), 137.3 (C-7a), 121.9 (C-4), 120.0 (C-3a), 112.2 (C-3a), 112.2 (C-5), 95.8 (C-7), 55.3 (C-17), 46.1 (C-15^a^), 44.7 (C-13^a^), 35.8 (C-11), 25.7 (C-12), 22.7 (C-16); (+)-HRESIMS *m/z* 605.3071 [M + H]^+^ (calcd for C_32_H_41_N_6_O_6_, 605.3082).

#### 3.2.13. *N*^1^,*N*^4^-Bis(3-(2-(7-methoxy-1*H*-indol-3-yl)acetamido)propyl)butane-1,4-diaminium 2,2,2-trifluoroacetate (**16**)

Using general procedure A, 2-(7-methoxy-1*H*-indol-3-yl)-2-oxoacetic acid (**12**) (110 mg, 0.50 mmol), spermine (48 mg, 0.24 mmol), PyBOP (261 mg, 0.50 mmol) and Et_3_N (417 μL, 3.0 mmol) afforded **16** as a yellow gum (67 mg, 49% yield).

R*_f_* = 0.03 (MeOH:TEA 5:0.01); IR ν_max_ (ATR) 3191, 1671, 1623, 1432, 1179, 785 cm^−1^; ^1^H NMR (DMSO-*d*_6_, 400 MHz) δ_H_12.45 (1H, s, NH-1), 8.89 (1H, t, *J* = 6.1 Hz, NH-10), 8.62 (1H, d, *J* = 3.4 Hz, H-2), 8.52 (1H, br s, NH-14), 7.80 (1H, d, *J* = 8.1 Hz, H-4), 7.19 (1H, t, *J* = 8.1 Hz, H-5), 6.86 (1H, d, *J* = 8.1 Hz, H-6), 3.95 (3H, s, H_3_-17), 3.33–3.23 (2H, m, H_2_-11), 2.99–2.89 (4H, m, H_2_-13 and H_2_-15), 1.90–1.80 (2H, m, H_2_-12), 1.65–1.58 (2H, m, H_2_-16); ^13^C NMR (DMSO-*d*_6_, 100 MHz) δ_C_ 181.7 (C-8), 163.7 (C-9), 146.4 (C-7), 137.4 (C-2), 127.8 (C-3a), 126.1 (C-7a), 123.6 (C-5), 113.7 (C-4), 112.6 (C-3), 104.4 (C-6), 55.4 (C-17), 46.1 (C-15^a^), 44.7 (C-13^a^), 35.8 (C-11), 25.7 (C-12), 22.7 (C-16); (+)-HRESIMS *m/z* 605.3065 [M + H]^+^ (calcd for C_32_H_41_N_6_O_6_, 605.3082).

#### 3.2.14. Di-*tert*-butyl Octane-1,8-diylbis((3-(2-(1*H*-indol-3-yl)-2-oxoacetamido)propyl)carbamate) (**17**)

Using general procedure A, 2-(1*H*-indol-3-yl)-2-oxoacetic acid (**9**) (109 mg, 0.58 mmol), di-*tert*-butyl octane-1,8-diylbis((3-aminopropyl)carbamate) [25] (120 mg, 0.26 mmol), PyBOP (300 mg, 0.58 mmol) and Et_3_N (218 μL, 1.5 mmol) afforded **17** as a white gum (34 mg, 16% yield).

R*_f_* = 0.66 (CH_2_Cl_2_:EtOAc 1:1); IR ν_max_ (ATR) 3215, 2925, 1618, 1420, 1152, 746 cm^−1^; ^1^H NMR (DMSO-*d*_6_, 300 MHz) δ 12.20 (1H, s, NH-1), 8.75 (1H, d, *J* = 3.0 Hz, H-2), 8.71 (1H, t, *J* = 6.0 Hz, NH-10), 8.26–8.19 (1H, m, H-4), 7.57–7.49 (1H, m, H-7), 7.30–7.20 (2H, m, H-5 and H-6), 3.25–3.05 (6H, m, H_2_-11, H_2_-13 and H_2_-15), 1.78–1.62 (2H, m, H_2_-12), 1.49–1.31 (2H, m, H_2_-16), 1.37 (9H, s, 3H_3_-21), 1.27–1.11 (4H, m, H_2_-17 and H_2_-18); ^13^C NMR (DMSO-*d*_6_, 100 MHz) δ_C_ 182.1 (C-8), 163.5 (C-9), 155.6 (C-19), 138.4 (C-2), 136.2 (C-7a), 126.2 (C-3a), 123.4 (C-5^a^), 122.5 (C-6^a^), 121.2 (C-4), 112.5 (C-3), 112.1 (C-7), 78.2 (C-20), 46.4 (C-15), 44.4, 44.0 (C-13), 36.3 (C-11), 28.7 (C-18), 28.0 (C-21), 27.8 (C-16 and C-12), 26.1 (C-17); (+)-HRESIMS *m/z* 801.4510 [M + H]^+^ (calcd for C_44_H_61_N_6_O_8_, 801.4545).

#### 3.2.15. Di-*tert*-butyl Octane-1,8-diylbis((3-(2-(5-methoxy-1*H*-indol-3-yl)-2-oxoacetamido)propyl)carbamate) (**18**)

Using general procedure A, 2-(5-methoxy-1*H*-indol-3-yl)-2-oxoacetic acid (**10**) (93 mg, 0.42 mmol), di-*tert*-butyl octane-1,8-diylbis((3-aminopropyl)carbamate) [25] (97 mg, 0.21 mmol), PyBOP (242 mg, 0.47 mmol) and Et_3_N (176 μL, 1.3 mmol) afforded **18** as a yellow oil (83 mg, 46% yield).

R*_f_* = 0.39 (hexane:EtOAc 2:3); IR ν_max_ (ATR) 3371, 2929, 1619, 1420, 1153, 736 cm^−1^; ^1^H NMR (DMSO-*d*_6_, 300 MHz) δ_H_ 12.08 (1H, s, NH-1), 8.69 (1H, d, *J* = 2.3 Hz, H-2), 8.67 (1H, m, NH-10), 7.74 (1H, d, *J* = 2.6 Hz, H-4), 7.42 (1H, d, *J* = 8.1 Hz, H-7), 6.89 (1H, dd, *J* = 8.1, 2.6 Hz, H-6), 3.79 (3H, s, H_3_-19), 3.24–3.03 (6H, m, H_2_-11, H_2_-13 and H_2_-15), 1.77–1.62 (2H, m, H_2_-12), 1.49–1.32 (2H, m, H_2_-16), 1.37 (9H, s, 3H_3_-22), 1.27–1.11 (4H, m, H_2_-17 and H_2_-18); ^13^C NMR (DMSO-*d*_6_, 75 MHz) δ_C_ 181.9 (C-8), 163.6 (C-9), 155.9 (C-5), 154.6 (C-20), 138.4 (C-2), 131.0 (C-7a), 127.2 (C-3a), 113.2 (C-6), 112.8 (C-7), 112.0 (C-3), 103.4 (C-4), 78.2 (C-21), 55.2 (C-19), 46.3 (C-15), 44.3, 44.0 (C-13), 36.3 (C-11), 28.7 (C-18), 28.0 (C-22), 27.8 (C-16 and C-12), 26.1 (C-17); (+)-HRESIMS *m/z* 861.4725 [M + H]^+^ (calcd for C_46_H_65_N_6_O_10_, 861.4757).

#### 3.2.16. Di-*tert*-butyl Octane-1,8-diylbis((3-(2-(6-methoxy-1*H*-indol-3-yl)-2-oxoacetamido)propyl)carbamate) (**19**)

Using general procedure A, 2-(6-methoxy-1*H*-indol-3-yl)-2-oxoacetic acid (**11**) (94 mg, 0.43 mmol), di-*tert*-butyl octane-1,8-diylbis((3-aminopropyl)carbamate) [25] (98 mg, 0.21 mmol), PyBOP (245 mg, 0.47 mmol) and Et_3_N (178 μL, 1.3 mmol) afforded **19** as a creamy solid (92 mg, 50% yield).

Mp 92 °C ; R*_f_* = 0.23 (CH_2_Cl_2_:EtOAc 1:1); IR ν_max_ (ATR) 3329, 2933, 1612, 1423, 1159, 740 cm^−1^; ^1^H NMR (DMSO-*d*_6_, 300 MHz) δ_H_ 11.99 (1H, d, *J* = 2.8 Hz, NH-1), 8.68 (1H, t, *J* = 5.7 Hz, NH-10), 8.64 (1H, d, *J* = 2.8 Hz, H-2), 8.07 (1H, d, *J* = 8.8 Hz, H-4), 7.02 (1H, d, *J* = 2.2 Hz, H-7), 6.88 (1H, dd, *J* = 8.8, 2.2 Hz, H-5), 3.79 (3H, s, H_3_-19), 3.23–3.05 (6H, m, H_2_-11, H_2_-13 and H_2_-15), 1.77–1.63 (2H, m, H_2_-12), 1.48–1.32 (2H, m, H_2_-16), 1.36 (9H, s, 3H_3_-22), 1.28–1.13 (4H, m, H_2_-17 and H_2_-18); ^13^C NMR (DMSO-*d*_6_, 75 MHz) δ_C_ 181.9 (C-8), 163.5 (C-9), 156.7 (C-6), 154.6 (C-20), 137.6 (C-2), 137.2 (C-7a), 121.9 (C-4), 120.0 (C-3a), 112.3 (C-3), 112.0 (C-5), 95.7 (C-7), 78.2 (C-21), 55.2 (C-19), 46.3 (C-15), 44.3, 44.0 (C-13), 36.3 (C-11), 28.7 (C-18), 28.0 (C-22), 27.7 (C-16 and C-12), 26.1 (C-17); (+)-HRESIMS *m/z* 861.4743 [M + H]^+^ (calcd for C_46_H_65_N_6_O_10_, 861.4757).

#### 3.2.17. Di-*tert*-butyl Octane-1,8-diylbis((3-(2-(7-methoxy-1*H*-indol-3-yl)-2-oxoacetamido)propyl)carbamate) (**20**)

Using general procedure A, 2-(7-methoxy-1*H*-indol-3-yl)-2-oxoacetic acid (**12**) (86 mg, 0.39 mmol), di-*tert*-butyl octane-1,8-diylbis((3-aminopropyl)carbamate) [25] (90 mg, 0.20 mmol), PyBOP (225 mg, 0.43 mmol) and Et_3_N (163 μL, 1.2 mmol) afforded **20** as a green gum (94 mg, 56% yield).

R*_f_* = 0.57 (CH_2_Cl_2_:EtOAc 1:1) 0.57; IR ν_max_ (ATR) 3366, 2933, 1617, 1455, 1160, 778 cm^−1^; ^1^H NMR (DMSO-*d*_6_, 400 MHz) δ_H_ 12.39 (1H, br d, *J* = 3.1 Hz, NH-1), 8.71 (1H, br t, *J* = 5.0 Hz, NH-10), 8.61 (1H, d, *J* = 3.1 Hz, H-2), 7.80 (1H, d, *J* = 7.8 Hz, H-4), 7.17 (1H, t, *J* = 7.8 Hz, H-5), 6.85 (1H, d, *J* = 7.8 Hz, H-6), 3.94 (3H, s, H_3_-19), 3.22–3.05 (6H, m, H_2_-11, H_2_-13 and H_2_-15), 1.76–1.64 (2H, m, H_2_-12), 1.47–1.37 (2H, m, H_2_-16), 1.36 (9H, s, 3H_3_-22), 1.27–1.12 (4H, m, H_2_-17 and H_2_-18); ^13^C NMR (DMSO-*d*_6_, 100 MHz) δ_C_ 182.1 (C-8), 163.4 (C-9), 154.7 (C-20), 146.4 (C-7), 137.3 (C-2), 127.8 (C-3a), 126.1 (C-7a), 123.4 (C-5), 113.8 (C-4), 112.7 (C-3), 104.3 (C-6), 78.2 (C-21), 55.4 (C-19), 46.3 (C-15), 44.4, 44.0 (C-13), 36.3 (C-11), 28.7 (C-18), 28.0 (C-22), 27.7 (C-16 and C-12), 26.1 (C-17); (+)-HRESIMS *m/z* 861.4778 [M + H]^+^ (calcd for C_46_H_65_N_6_O_10_, 861.4757).

#### 3.2.18. Di-*tert*-butyl Dodecane-1,12-diylbis((3-(2-(1*H*-indol-3-yl)-2-oxoacetamido)propyl)carbamate) (**21**)

Using general procedure A, 2-(1*H*-indol-3-yl)-2-oxoacetic acid (**9**) (19 mg, 0.10 mmol), di-*tert*-butyl dodecane-1,12-diylbis((3-aminopropyl)carbamate) [27,28] (23 mg, 45 μmol), PyBOP (51 mg, 0.10 mmol) and Et_3_N (82 μL, 0.60 mmol) afforded **21** as a white gum (25 mg, 65% yield).

R*_f_* = 0.60 (CH_2_Cl_2_:EtOAc 1:1); IR ν_max_ (ATR) 2927, 1621, 1420, 1156, 746 cm^−1^; ^1^H NMR (DMSO-*d*_6_, 400 MHz) δ_H_ 12.20 (1H, s, NH-1), 8.75 (1H, s, H-2), 8.72 (1H, t, *J* = 6.1 Hz, NH-10), 8.25–8.19 (1H, m, H-4), 7.56–7.50 (1H, m, H-7), 7.30–7.21 (2H, m, H-5 and H-6), 3.24–3.06 (6H, m, H_2_-11, H_2_-13 and H_2_-15), 1.77–1.64 (2H, m, H_2_-12), 1.49–1.33 (2H, m, H_2_-16), 1.37 (9H, s, 3H_3_-23), 1.25–1.16 (8H, m, H_2_-17 to H_2_-20); ^13^C NMR (DMSO-*d*_6_, 100 MHz) δ_C_ 182.1 (C-8), 163.5 (C-9), 155.0 (C-21), 138.4 (C-2), 136.2 (C-7a), 126.2 (C-3a), 123.4 (C-4), 122.5 (C-5^a^), 121.3 (C-6^a^), 112.5 (C-7), 112.2 (C-3), 78.2 (C-22), 46.3 (C-15), 44.0 (C-13), 36.3 (C-11), 28.9 (C-18^b^), 28.9 (C-19^b^), 28.7 (C-20^b^), 28.3 (C-16), 28.0 (C-23), 27.7 (C-12), 26.1 (C-17^b^); (+)-HRESIMS *m/z* 879.4967 [M + Na]^+^ (calcd for C_48_H_68_N_6_NaO_8_, 879.4991).

#### 3.2.19. Di-*tert*-butyl Dodecane-1,12-diylbis((3-(2-(5-methoxy-1*H*-indol-3-yl)-2-oxoacetamido)propyl)carbamate) (**22**)

Using general procedure A, 2-(5-methoxy-1*H*-indol-3-yl)-2-oxoacetic acid (**10**) (50 mg, 0.23 mmol), di-*tert*-butyl dodecane-1,12-diylbis((3-aminopropyl)carbamate) [27,28] (53 mg, 0.10 mmol), PyBOP (117 mg, 0.23 mmol) and Et_3_N (86 μL, 0.62 mmol) afforded **22** as an orange gum (53 mg, 58% yield).

R*_f_* = 0.54 (hexane:EtOAc 3:7); IR ν_max_ (ATR) 3237, 2927, 1621, 1420, 1139, 735 cm^−1^; ^1^H NMR (DMSO-*d*_6_, 400 MHz) δ_H_ 12.09 (1H, s, NH-1), 8.69 (1H, d, *J* = 3.0 Hz, H-2), 8.67 (1H, m, NH-10), 7.74 (1H, d, *J* = 2.5 Hz, H-4), 7.42 (1H, d, *J* = 8.8 Hz, H-7), 6.89 (1H, dd, *J* = 8.8, 2.5 Hz, H-6), 3.79 (3H, s, H_3_-21), 3.23–3.05 (6H, m, H_2_-11, H_2_-13 and H_2_-15), 1.75–1.64 (2H, m, H_2_-12), 1.47–1.32 (2H, m, H_2_-16), 1.37 (9H, s, 3H_3_-24), 1.25–1.12 (8H, m, H_2_-17 to H_2_-20); ^13^C NMR (DMSO-*d*_6_, 100 MHz) δ_C_ 181.9 (C-8), 163.6 (C-9), 155.9 (C-5), 154.7 (C-22), 138.4 (C-2), 131.0 (C-7a), 127.2 (C-3a), 113.2 (C-7), 112.8 (C-6), 112.0 (C-3), 103.4 (C-4), 78.2 (C-23), 55.2 (C-21), 46.3 (C-15), 44.4, 44.0 (C-13), 36.3 (C-11), 28.9 (C-18^a^), 28.9 (C-19^a^), 28.6 (C-20^a^), 28.0 (C-24), 27.7 (C-12 and C-16), 26.2 (C-17^a^); (+)-HRESIMS *m/z* 917.5363 [M + H]^+^ (calcd for C_50_H_73_N_6_O_10_, 917.5383).

#### 3.2.20. Di-*tert*-butyl Dodecane-1,12-diylbis((3-(2-(6-methoxy-1*H*-indol-3-yl)-2-oxoacetamido)propyl)carbamate) (**23**)

Using general procedure A, 2-(6-methoxy-1*H*-indol-3-yl)-2-oxoacetic acid (**11**) (33 mg, 0.15 mmol), di-*tert*-butyl dodecane-1,12-diylbis((3-aminopropyl)carbamate) [27,28] (35 mg, 68 μmol), PyBOP (78 mg, 0.15 mmol) and Et_3_N (57 μL, 0.41 mmol) afforded **23** as a creamy gum (35 mg, 58% yield).

R*_f_* = 0.47 (EtOAc); IR ν_max_ (ATR) 3641, 2929, 1625, 1421, 1150, 831 cm^−1^; ^1^H NMR (DMSO-*d*_6_, 400 MHz) δ_H_11.99 (1H, br d, *J* = 2.5 Hz, NH-1), 8.68 (1H, t, *J* = 5.5 Hz, NH-10), 8.63 (1H, d, *J* = 2.5 Hz, H-2), 8.07 (1H, d, *J* = 8.8 Hz, H-4), 7.02 (1H, d, *J* = 2.3 Hz, H-7), 6.88 (1H, dd, *J* = 8.8, 2.3 Hz, H-5), 3.79 (3H, s, H_3_-21), 3.23–3.06 (6H, m, H_2_-11, H_2_-13 and H_2_-15), 1.76–1.63 (2H, m, H_2_-12), 1.47–1.33 (2H, m, H_2_-16), 1.37 (9H, s, 3H_3_-22), 1.25–1.12 (8H, m, H_2_-17, H_2_-18, H_2_-19 and H_2_-20); ^13^C NMR (DMSO-*d*_6_, 100 MHz) δ_C_181.4 (C-8), 163.0 (C-9), 156.2 (C-6), 154.2 (C-20), 137.2 (C-2), 136.7 (C-7a), 121.4 (C-4), 119.5 (C-3a), 111.8 (C-3), 111.6 (C-5), 95.2 (C-7), 77.8 (C-21), 54.8 (C-21), 45.8 (C-15), 43.9, 43.5 (C-13), 35.8 (C-11), 28.5 (C-18^a^), 28.4 (C-19^a^), 28.2 (C-20^a^), 27.5 (C-24), 27.2 (C-12 and C-16), 25.7 (C-17^a^); (+)-HRESIMS *m/z* 939.5161 [M + Na]^+^ (calcd for C_50_H_72_N_6_NaO_10_, 939.5202).

#### 3.2.21. Di-*tert*-butyl Dodecane-1,12-diylbis((3-(2-(7-methoxy-1*H*-indol-3-yl)-2-oxoacetamido)propyl)carbamate) (**24**)

Using general procedure A, 2-(7-methoxy-1*H*-indol-3-yl)-2-oxoacetic acid (**12**) (45 mg, 0.21 mmol), di-*tert*-butyl dodecane-1,12-diylbis((3-aminopropyl)carbamate) [27,28] (48 mg, 93 μmol), PyBOP (107 mg, 0.21 mmol) and Et_3_N (78 μL, 0.56 mmol) afforded **24** as a yellow oil (48 mg, 58% yield).

R*_f_* = 0.66 (hexane:EtOAc 3:7); IR ν_max_ (ATR) 3233, 2927, 1623, 1420, 1157, 782 cm^−1^; ^1^H NMR (DMSO-*d*_6_, 400 MHz) δ_H_ 12.39 (1H, s, NH-1), 8.71 (1H, br s, NH-10), 8.61 (1H, s, H-2), 7.80 (1H, d, *J* = 7.9 Hz, H-4), 7.17 (1H, t, *J* = 7.9 Hz, H-5), 6.84 (1H, d, *J* = 7.9 Hz, H-6), 3.94 (3H, s, H_3_-21), 3.22–3.06 (6H, m, H_2_-11, H_2_-13 and H_2_-15), 1.75–1.64 (2H, m, H_2_-12), 1.47–1.34 (2H, m, H_2_-16), 1.37 (9H, s, 3H_3_-24), 1.25–1.10 (8H, m, H_2_-17, H_2_-18, H_2_-19 and H_2_-20); ^13^C NMR (DMSO-*d*_6_, 100 MHz) δ_C_ 182.0 (C-8), 163.4 (C-9), 154.8 (C-22), 146.4 (C-7), 137.3 (C-2), 127.8 (C-3a), 126.1 (C-7a), 123.4 (C-5), 113.8 (C-4), 112.7 (C-3), 104.3 (C-6), 78.2 (C-23), 55.3 (C-21), 46.3 (C-15), 44.4, 44.0 (C-13), 36.3 (C-11), 28.9 (C-18^a^), 28.9 (C-19^a^), 28.6 (C-20^a^), 28.0 (C-24), 27.7 (C-12 and C-16), 26.2 (C-17^a^); (+)-HRESIMS *m/z* 917.5369 [M + H]^+^ (calcd for C_50_H_73_N_6_O_10_, 917.5383). 

#### 3.2.22. *N*^1^,*N*^8^-Bis(3-(2-(1*H*-indol-3-yl)-2-oxoacetamido)propyl)octane-1,8-diaminium 2,2,2-trifluoroacetate (**25**)

Using general procedure B, reaction of **17** (12 mg, 15 μmol) in CH_2_Cl_2_ (1.7 mL) with TFA (0.3 mL) followed by purification by C_18_ reversed-phase column chromatography (30% MeOH/H_2_O (TFA)) afforded **25** as a yellow oil (12 mg, quant. yield).

R*_f_* = 0.23 (CH_2_Cl_2_:MeOH:TEA 4:1:0.01); IR ν_max_ (ATR) 3235, 1669, 1431, 1200, 1130, 721 cm^−1^; ^1^H NMR (DMSO-*d*_6_, 400 MHz) δ_H_ 12.29 (1H, s, NH-1), 8.88 (1H, t, *J* = 6.2 Hz, NH-10), 8.76 (1H, s, H-2), 8.26–8.20 (1H, m, H-4), 7.57–7.51 (1H, m, H-7), 7.30–7.23 (2H, m, H-5 and H-6), 3.30 (2H, t, *J* = 6.2 Hz, H_2_-11), 2.98–2.91 (2H, m, H_2_-13), 2.91–2.84 (2H, m, H_2_-15), 1.91–1.80 (2H, m, H_2_-12), 1.61–1.50 (2H, m, H_2_-16), 1.35–1.21 (4H, m, H_2_-17 and H_2_-18); ^13^C NMR (DMSO-*d*_6_, 100 MHz) δ_C_ 181.7 (C-8), 163.7 (C-9), 138.3 (C-7a), 136.2 (C-2), 126.3 (C-3a), 123.6 (C-5^a^), 122.7 (C-6^a^), 121.3 (C-4), 112.6 (C-7), 112.2 (C-3), 46.7 (C-15), 44.6 (C-13), 35.7 (C-11), 28.4 (C-18), 25.9 (C-12^b^), 25.7 (C-17^b^), 22.8 (C-16^b^); (+)-HRESIMS *m/z* 601.3488 [M + H]^+^ (calcd for C_34_H_45_N_6_O_4_, 601.3497).

#### 3.2.23. *N*^1^,*N*^8^-Bis(3-(2-(5-methoxy-1*H*-indol-3-yl)-2-oxoacetamido)propyl)octane-1,8-diaminium 2,2,2-trifluoroacetate (**26**)

Using general procedure B, reaction of **18** (27 mg, 31 μmol) in CH_2_Cl_2_ (1.7 mL) with TFA (0.3 mL) afforded **26** as a brown gum (20 mg, 96% yield) which required no further purification.

R*_f_* = 0.20 (CH_2_Cl_2_:MeOH:TEA 4:1:0.01); IR ν_max_ (ATR) 3407, 1674, 1478, 1181, 1025, 723 cm^−1^; ^1^H NMR (CD_3_OD, 400 MHz) δ_H_8.73 (1H, s, H-2), 7.84 (1H, d, *J* = 2.5 Hz, H-4), 7.38 (1H, d, *J* = 8.8 Hz, H-7), 6.91 (1H, dd, *J* = 8.8, 2.5 Hz, H-6), 3.85 (3H, s, H_3_-19), 3.49–3.43 (2H, t, *J* = 6.5 Hz, H_2_-11), 3.08–3.02 (2H, m, H_2_-13), 3.01–2.95 (2H, m, H_2_-15), 1.99 (2H, tt, *J* = 7.1, 6.5 Hz, H_2_-12), 1.73–1.63 (2H, m, H_2_-16), 1.44–1.33 (4H, m, H_2_-17 and H_2_-18); ^13^C NMR (CD_3_OD, 100 MHz) δ_C_ 182.0 (C-8), 166.5 (C-9), 158.2 (C-5), 139.6 (C-2), 132.7 (C-7a), 128.9 (C-3a), 114.6 (C-6), 113.9 (C-3 and C-7), 105.1 (C-4), 56.1 (C-19), 48.8 (C-15), 46.4 (C-13), 36.9 (C-11), 29.9 (C-18^a^), 27.4 (C-12^a^), 27.4 (C-17^a^), 27.2 (C-16^a^); (+)-HRESIMS *m/z* 661.3690 [M + H]^+^ (calcd for C_36_H_49_N_6_O_6_, 661.3708).

#### 3.2.24. *N*^1^,*N*^8^-Bis(3-(2-(6-methoxy-1*H*-indol-3-yl)-2-oxoacetamido)propyl)octane-1,8-diaminium 2,2,2-trifluoroacetate (**27**)

Using general procedure B, reaction of **19** (11 mg, 13 μmol) in CH_2_Cl_2_ (1.7 mL) with TFA (0.3 mL) afforded **27** as a yellow oil (5 mg, 59% yield) which required no further purification.

R*_f_* = 0.19 (CH_2_Cl_2_:MeOH:TEA 4:1:0.01); IR ν_max_ (ATR) 3395, 1671, 1150, 1199, 1022, 722 cm^−1^; ^1^H NMR (CD_3_OD, 400 MHz) δ_H_ 8.67 (1H, s, H-2), 8.15 (1H, d, *J* = 8.8 Hz, H-4), 7.01 (1H, d, *J* = 2.4 Hz, H-7), 6.90 (1H, dd, *J* = 8.8, 2.4 Hz, H-6), 3.84 (3H, s, H_3_-19), 3.45 (2H, t, *J* = 6.6 Hz, H_2_-11), 3.05 (2H, t, *J* = 7.6, H_2_-13), 3.02–2.96 (2H, m, H_2_-15), 1.98 (2H, tt, *J* = 7.6, 6.6 Hz, H_2_-12), 1.73–1.63 (2H, m, H_2_-16), 1.45–1.35 (4H, m, H_2_-17 and H_2_-18); ^13^C NMR (CD_3_OD, 100 MHz) δ_C_ 182.0 (C-8), 166.5 (C-9), 159.1 (C-6), 139.0 (C-7a), 138.9 (C-2), 123.6 (C-4), 121.7 (C-3a), 114.1 (C-3), 113.5 (C-5), 96.5 (C-7), 56.0 (C-19), 49.2 (C-15), 46.5 (C-13), 36.9 (C-11), 30.0 (C-18), 27.5 (C-12^a^), 27.5 (C-17^a^), 27.3 (C-16^a^); (+)-HRESIMS *m/z* 661.3687 [M + H]^+^ (calcd for C_36_H_49_N_6_O_6_, 661.3708).

#### 3.2.25. *N*^1^,*N*^8^-Bis(3-(2-(7-methoxy-1*H*-indol-3-yl)-2-oxoacetamido)propyl)octane-1,8-diaminium 2,2,2-trifluoroacetate (**28**)

Using general procedure B, reaction of **20** (20 mg, 13 μmol) in CH_2_Cl_2_ (1.8 mL) with TFA (0.2 mL) afforded **28** as a yellow oil (12 mg, quant. yield) which required no further purification.

R*_f_* = 0.26 (CH_2_Cl_2_:MeOH:TEA 4:1:0.01); IR ν_max_ (ATR) 3337, 2941, 1622, 1432, 1132, 721 cm^−1^; ^1^H NMR (CD_3_OD, 400 MHz) δ_H_ 8.70 (1H, br d, *J* = 1.0 Hz, H-2), 7.86 (1H, d, *J* = 8.2 Hz, H-4), 7.18 (1H, t, *J* = 8.2 Hz, H-5), 6.81 (1H, d, *J* = 8.2 Hz, H-6), 3.97 (3H, s, H_3_-19), 3.45 (2H, t, *J* = 6.5 Hz, H_2_-11), 3.04 (2H, t, *J* = 7.1 Hz, H_2_-13), 2.97 (2H, t, *J* = 8.0 Hz, H_2_-15), 1.98 (2H, tt, *J* = 7.1, 6.5 Hz, H_2_-12), 1.72–1.62 (2H, m, H_2_-16), 1.37–1.23 (4H, m, H_2_-17 and H_2_-18); ^13^C NMR (CD_3_OD, 100 MHz) δ_C_182.2 (C-8), 166.4 (C-9), 148.1 (C-7), 138.6 (C-2), 129.5 (C-3a), 128.0 (C-7a), 124.8 (C-5), 115.4 (C-4), 114.4 (C-3), 105.3 (C-6), 56.0 (C-19), 49.2 (C-15), 46.4 (C-13), 36.9 (C-11), 29.9 (C-18^a^), 27.4 (C-12^a^), 27.4 (C-17^a^), 27.2 (C-16^a^); (+)-HRESIMS *m/z* 661.3695 [M + H]^+^ (calcd for C_36_H_49_N_6_O_6_, 661.3708).

#### 3.2.26. *N*^1^,*N*^12^-Bis(3-(2-(1*H*-indol-3-yl)-2-oxoacetamido)propyl)dodecane-1,12-diaminium 2,2,2-trifluoroacetate (**29**)

Using general procedure B, reaction of **21** (14 mg, 16 μmol) in CH_2_Cl_2_ (1.8 mL) with TFA (0.2 mL) afforded **29** as a white gum (5 mg, 47% yield) which required no further purification.

R*_f_* = 0.26 (CH_2_Cl_2_:MeOH:TEA 4:1:0.01); IR ν_max_ (ATR) 3391, 2949, 1675, 1434, 1132, 1034, 722 cm^−1^; ^1^H NMR (CD_3_OD, 400 MHz) δ_H_ 8.80 (1H, d, *J* = 1.7 Hz, H-2), 8.34–8.28 (1H, m, H-4), 7.52–7.46 (1H, m, H-7), 7.31–7.23 (2H, m, H-5 and H-6), 3.51–3.42 (2H, m, H_2_-11), 3.11–3.03 (2H, m, H_2_-13), 3.03–2.95 (2H, m, H_2_-15), 2.05–1.93 (2H, m, H_2_-12), 1.74–1.62 (2H, m, H_2_-16), 1.44–1.23 (8H, m, H_2_-17 to H_2_-20); ^13^C NMR (CD_3_OD, 100 MHz) δ_C_ 182.0 (C-8), 166.4 (C-9), 139.6 (C-2), 138.0 (C-7a), 127.9 (C-3a), 124.9 (C-5^a^), 123.9 (C-6^a^), 123.0 (C-4), 114.0 (C-7), 113.2 (C-3), 48.6 (C-15), 46.4 (C-13), 36.9 (C-11), 30.6 (C-18^b^), 30.5 (C-19^b^), 30.2 (C-20^b^), 27.5 (C-12^b^), 27.5 (C-17^b^), 27.3 (C-16^b^); (+)-HRESIMS *m/z* 329.2098 [M + 2H]^2+^ (calcd for C_38_H_54_N_6_O_4_, 329.2098).

#### 3.2.27. *N*^1^,*N*^12^-Bis(3-(2-(5-methoxy-1*H*-indol-3-yl)-2-oxoacetamido)propyl)dodecane-1,12-diaminium 2,2,2-trifluoroacetate (**30**)

Using general procedure B, reaction of **22** (11 mg, 12 μmol) in CH_2_Cl_2_ (1.8 mL) with TFA (0.2 mL) afforded **30** as a yellow gum (8 mg, 90% yield) which required no further purification.

R*_f_* = 0.29 (CH_2_Cl_2_:MeOH:TEA 4:1:0.01); IR ν_max_ (ATR) 3033, 2930, 1670, 1618, 1434, 1178, 1130, 721 cm^−1^; ^1^H NMR (CD_3_OD, 400 MHz) δ_H_ 8.74 (1H, s, H-2), 7.85 (1H, d, *J* = 2.3 Hz, H-4), 7.38 (1H, d, *J* = 8.8 Hz, H-7), 6.91 (1H, dd, J = 8.8, 2.3 Hz, H-6), 3.85 (3H, s, H_3_-21), 3.46 (2H, t, *J* = 6.4 Hz, H_2_-11), 3.06 (2H, t, *J* = 7.2 Hz, H_2_-13), 3.00 (2H, t, *J* = 7.6 Hz, H_2_-15), 1.99 (2H, tt, *J* = 7.2, 6.4 Hz, H_2_-12), 1.74–1.63 (2H, m, H_2_-16), 1.43–1.25 (8H, m, H_2_-17 to H_2_-20); ^13^C NMR (CD_3_OD, 100 MHz) δ_C_ 181.9 (C-8), 166.5 (C-9), 158.2 (C-5), 139.6 (C-2), 132.7 (C-7a), 128.9 (C-3a), 114.6 (C-6), 113.9 (C-3 and C-7), 105.1 (C-4), 56.1 (C-21), 49.0 (C-15), 46.4 (C-13), 36.9 (C-11), 30.6 (C-18^a^), 30.5 (C-19^a^), 30.2 (C-20^a^), 27.5 (C-12^b^) 27.5 (C-17^b^), 27.3 (C-16); (+)-HRESIMS *m/z* 717.4304 [M + H]^+^ (calcd for C_40_H_57_N_6_O_6_, 717.4334).

#### 3.2.28. *N*^1^,*N*^12^-Bis(3-(2-(6-methoxy-1*H*-indol-3-yl)-2-oxoacetamido)propyl)dodecane-1,12-diaminium 2,2,2-trifluoroacetate (**31**)

Using general procedure B, reaction of **23** (14 mg, 16 μmol) in CH_2_Cl_2_ (1.8 mL) with TFA (0.2 mL) afforded **31** as a yellow gum (16 mg, quant. yield) which required no further purification.

R*_f_* = 0.31 (CH_2_Cl_2_:MeOH:TEA 4:1:0.01); IR ν_max_ (ATR) 3346, 1626, 1449, 1153, 518 cm^−1^; ^1^H NMR (CD_3_OD, 400 MHz) δ_H_8.70 (1H, s, H-2), 8.17 (1H, d, *J* = 8.7 Hz, H-4), 7.05 (1H, d, *J* = 2.3 Hz, H-7), 6.93 (1H, dd, *J* = 8.7, 2.3 Hz, H-6), 3.87 (3H, s, H_3_-21), 3.49 (2H, t, *J* = 6.6 Hz, H_2_-11), 3.08 (2H, t, *J* = 7.5, H_2_-13), 3.02 (2H, t, *J* = 7.6 Hz, H_2_-15), 2.02 (2H, tt, *J* = 7.5, 6.6 Hz, H_2_-12), 1.76–1.66 (2H, m, H_2_-16), 1.47–1.23 (4H, m, H_2_-17 to H_2_-20); ^13^C NMR (CD_3_OD, 100 MHz) δ_C_ 182.0 (C-8), 166.4 (C-9), 159.0 (C-6), 139.2 (C-7a), 139.0 (C-2), 123.5 (C-6), 121.7 (C-3a), 114.0 (C-3), 113.5 (C-5), 96.6 (C-7), 56.1 (C-21), 49.0 (C-15), 46.4 (C-13), 36.9 (C-11), 30.6 (C-18^a^), 30.4 (C-19^a^), 30.2 (C-20^a^), 27.5 (C-12^a^), 27.4 (C-17^a^), 27.2 (C-16^a^); (+)-HRESIMS *m/z* 717.4327 [M + H]^+^ (calcd for C_40_H_57_N_6_O_6_, 717.4334).

#### 3.2.29. *N*^1^,*N*^12^-Bis(3-(2-(7-methoxy-1*H*-indol-3-yl)-2-oxoacetamido)propyl)dodecane-1,12-diaminium 2,2,2-trifluoroacetate (**32**)

Using general procedure B, reaction of **24** (8 mg, 9.0 μmol) in CH_2_Cl_2_ (1.8 mL) with TFA (0.2 mL) afforded **32** as a yellow oil (5 mg, 77% yield) which required no further purification.

R*_f_* = 0.43 (CH_2_Cl_2_:MeOH:TEA 4:1:0.01); IR ν_max_ (ATR) 3408, 1670, 1623, 1432, 1135, 737 cm^−1^; ^1^H NMR (CD_3_OD, 400 MHz) δ_H_ 8.71 (1H, s, H-2), 7.87 (1H, d, *J* = 7.9 Hz, H-4), 7.19 (1H, t, *J* = 7.9 Hz, H-5), 6.82 (1H, d, *J* = 7.9 Hz, H-6), 3.98 (3H, s, H_3_-21), 3.46 (2H, t, *J* = 6.2 Hz, H_2_-11), 3.05 (2H, t, *J* = 7.9 Hz, H_2_-13), 2.99 (2H, t, *J* = 8.4 Hz, H_2_-15), 2.02–1.93 (2H, m, H_2_-12), 1.73–1.63 (2H, m, H_2_-16), 1.43–1.26 (8H, m, H_2_-17, H_2_-18, H_2_-19 and H_2_-18); ^13^C NMR (CD_3_OD, 100 MHz) δ_C_ 182.1 (C-8), 166.4 (C-9), 148.1 (C-7), 138.6 (C-2), 129.5 (C-3a), 128.0 (C-7a), 124.8 (C-5), 115.4 (C-4), 114.4 (C-3), 105.3 (C-6), 56.0 (C-21), 47.9 (C-15), 46.4 (C-13), 36.9 (C-11), 30.6 (C-18^a^), 30.5 (C-19^a^), 30.2 (C-20^a^), 27.5 (C-12^a^), 27.5 (C-17^a^), 27.3 (C-16^a^); (+)-HRESIMS *m/z* 717.4326 [M + H]^+^ (calcd for C_40_H_57_N_6_O_6_, 717.4334).

#### 3.2.30. Di-*tert*-butyl Butane-1,4-diylbis((3-(2-(1*H*-indol-3-yl)acetamido)propyl)carbamate) (**33**)

Using general procedure A, 2-(1*H*-indol-3-yl)acetic acid [26] (40 mg, 0.23 mmol), di-*tert*-butyl butane-1,4-diylbis((3-aminopropyl)carbamate) [25,27] (42 mg, 0.10 mmol), PyBOP (119 mg, 0.23 mmol) and Et_3_N (87 μL, 0.63 mmol) afforded **33** as a yellow oil (29 mg, 39% yield).

R*_f_* = 0.14 (EtOAc); IR ν_max_ (ATR) 3320, 2942, 1660, 1421, 1162, 1025, 742 cm^−1^; ^1^H NMR (DMSO-*d*_6_, 400 MHz) δ_H_10.84 (1H, s, NH-1), 7.83 (1H, t, *J* = 5.6 Hz, NH-10), 7.54 (1H, d, *J* = 8.1 Hz, H-4), 7.33 (1H, d, *J* = 8.3 Hz, H-7), 7.17 (1H, d, *J* = 2.3 Hz, H-2), 7.05 (1H, ddd, *J* = 8.6, 8.3, 1.0 Hz, H-6), 6.95 (1H, ddd, *J* = 8.6, 8.1, 1.0 Hz, H-5), 3.48 (2H, s, H_2_-8), 3.13–2.96 (6H, m, H_2_-11, H_2_-13 and H_2_-15), 1.64–1.51 (2H, m, H_2_-12), 1.36 (9H, s, 3H_3_-19), 1.33–1.27 (2H, m, H_2_-16); ^13^C NMR (DMSO-*d*_6_, 100 MHz) δ_C_ 170.6 (C-9), 154.6 (C-17), 136.1 (C-7a), 127.2 (C-3a), 123.7 (C-2), 120.9 (C-6), 118.6 (C-4), 118.2 (C-5), 111.3 (C-7), 108.9 (C-3), 78.2 (C-18), 46.5, 46.1 (C-15), 44.6, 44.4 (C-13), 36.4 (C-11), 32.8 (C-8), 28.8 (C-12), 28.0 (C-19), 25.6, 25.1 (C-16); (+)-HRESIMS *m/z* 717.4310 [M + H]^+^ (calcd for C_40_H_57_N_6_O_6_, 717.4334).

#### 3.2.31. Di-*tert*-butyl Octane-1,8-diylbis((3-(2-(1*H*-indol-3-yl)acetamido)propyl)carbamate) (**34**)

To a stirred solution of 2-(1*H*-indol-3-yl)acetic acid [26] (51 mg, 0.29 mmol), DIPEA (68 μL, 0.41 mmol) in DMF (1 mL) was added HATU (110 mg, 0.29 mmol). The reaction mixture was stirred under N_2_ at r.t. for 80 min, followed by the addition of di-*tert*-butyl octane-1,8-diylbis((3-aminopropyl)carbamate) [25] (63 mg, 0.14 mmol). The reaction mixture was further stirred for 22 h and then partitioned between H_2_O (30 mL) and CH_2_Cl_2_ (3 × 40 mL). The combined organic extracts were washed with brine (20 mL) and dried over MgSO_4_ and concentrated *in vacuo*. Purification by silica gel flash column chromatography (hexanes/EtOAc 1:1 to EtOAc/MeOH 4:1) afforded **34** as a yellow gum (79 mg, 35% yield).

R*_f_* = 0.46 (EtOAc); IR ν_max_ (ATR) 3283, 2930, 1658, 1419, 1156, 740 cm^−1^; ^1^H NMR (DMSO-*d*_6_, 400 MHz) δ_H_ 10.84 (1H, s, NH-1), 7.82 (1H, t, *J* = 5.6 Hz, NH-10), 7.53 (1H, d, *J* = 7.9 Hz, H-4), 7.33 (1H, m, H-7), 7.17 (1H, d, *J* = 2.1 Hz, H-2), 7.05 (1H, ddd, *J* = 8.1, 8.0, 1.0 Hz, H-6), 6.95 (1H, ddd, *J* = 8.1, 7.9, 1.0 Hz, H-5), 3.48 (2H, s, H_2_-8), 3.13–2.96 (6H, m, H_2_-11, H_2_-13 and H_2_-15), 1.63–1.52 (2H, m, H_2_-12), 1.44–1.33 (2H, m, H_2_-16), 1.36 (9H, s, 3H_3_-21), 1.26–1.19 (2H, m, H_2_-18), 1.17–1.11 (2H, m, H_2_-17); ^13^C NMR (DMSO-*d*_6_, 100 MHz) δ_C_ 170.6 (C-9), 154.6 (C-19), 136.1 (C-7a), 127.2 (C-3a), 123.7 (C-2), 120.8 (C-6), 118.6 (C-4), 118.2 (C-5), 111.3 (C-7), 108.9 (C-3), 78.2 (C-20), 46.4 (C-15), 44.6, 44.2 (C-13), 36.4 (C-11), 32.8 (C-8), 28.7 (C-18), 28.0 (C-21), 27.8 (C-16 and C-12), 26.1 (C-17); (+)-HRESIMS *m/z* 773.4937 [M + H]^+^ (calcd for C_44_H_65_N_6_O_6_, 773.4960).

#### 3.2.32. Di-*tert*-butyl Dodecane-1,12-diylbis((3-(2-(1*H*-indol-3-yl)acetamido)propyl)carbamate) (**35**)

Using general procedure A, 2-(1*H*-indol-3-yl)acetic acid [26] (58 mg, 0.33 mmol), di-*tert*-butyl dodecane-1,12-diylbis((3-aminopropyl)carbamate) [27,28] (78 mg, 0.15 mmol), PyBOP (174 mg, 0.33 mmol) and Et_3_N (126 μL, 0.91 mmol) afforded **35** as a yellow oil (55 mg, 44% yield).

R*_f_* = 0.60 (EtOAc); IR ν_max_ (ATR) 3279, 2925, 1659, 1417, 1155, 740 cm^−1^; ^1^H NMR (DMSO-*d*_6_, 400 MHz) δ_H_ 10.84 (1H, s, NH-1), 7.83 (1H, t, *J* = 5.5 Hz, NH-10), 7.54 (1H, d, *J* = 8.0 Hz, H-4), 7.33 (1H, d, *J* = 8.1 Hz, H-7), 7.17 (1H, d, *J* = 1.8 Hz, H-2), 7.05 (1H, t, *J* = 8.1 Hz, H-6), 6.95 (1H, t, *J* = 8.0 Hz, H-5), 3.48 (2H, s, H_2_-8), 3.14–2.96 (6H, m, H_2_-11, H_2_-13 and H_2_-15), 1.63–1.52 (2H, m, H_2_-12), 1.44–1.32 (2H, m, H_2_-16), 1.36 (9H, s, 3H_3_-23), 1.27–1.20 (6H, m, H_2_-18 to H_2_-20), 1.19–1.11 (2H, m, H_2_-17); ^13^C NMR (DMSO-*d*_6_, 100 MHz) δ_C_ 170.6 (C-9), 154.6 (C-21), 136.1 (C-7a), 127.2 (C-3a), 123.7 (C-2), 120.9 (C-6), 118.6 (C-4), 118.2 (C-5), 111.3 (C-7), 108.9 (C-3), 78.2 (C-22), 46.5 (C-15), 44.5, 44.2 (C-13), 36.4 (C-11), 32.8 (C-8), 29.0 (C-18^a^), 28.9 (C-19^a^), 28.7 (C-20^a^), 28.0 (C-23), 27.8 (C-12 and C-16), 26.2 (C-17); (+)-HRESIMS *m/z* 851.5418 [M + Na]^+^ (calcd for C_48_H_72_N_6_NaO_6_, 851.5406).

#### 3.2.33. *N*^1^,*N*^4^-Bis(3-(2-(1*H*-indol-3-yl)acetamido)propyl)butane-1,4-diaminium 2,2,2-trifluoroacetate (**36**)

Using general procedure B, reaction of **33** (10 mg, 14 μmol) in CH_2_Cl_2_ (1.7 mL) with TFA (0.3 mL) and subsequent purification by C_18_ reversed-phase column chromatography (30% MeOH/H_2_O (TFA)) afforded **36** as a red oil (6 mg, 83% yield).

R*_f_* = 0.09 (CH_2_Cl_2_:MeOH:TEA 1:1:0.01); IR ν_max_ (ATR) 3284, 1672, 1551, 1456, 1340, 1180, 721 cm^−1^; ^1^H NMR (DMSO-*d*_6_, 300 MHz) δ_H_ 10.89 (1H, s, NH-1), 8.40 (2H, br s, NH_2_-14), 8.07 (1H, t, *J* = 6.2 Hz, NH-10), 7.54 (1H, d, *J* = 8.1 Hz, H-4), 7.35 (1H, ddd, *J* = 8.0, 0.9, 0.7 Hz, H-7), 7.19 (1H, d, *J* = 2.2 Hz, H-2) 7.07 (1H, ddd, *J* = 8.0, 8.0, 1.2 Hz, H-6), 6.97 (1H, ddd, *J* = 8.1, 8.0, 0.9 Hz, H-5), 3.52 (2H, s, H_2_-8), 3.13 (2H, td, *J* = 6.9, 6.2 Hz, H_2_-11), 2.89–2.70 (4H, m, H_2_-13 and H_2_-15), 1.78–1.65 (2H, m, H_2_-12), 1.60–1.46 (2H, m, H_2_-16); ^13^C NMR (DMSO-*d*_6_, 75 MHz) δ_C_ 171.5 (C-9), 136.1 (C-7a), 127.1 (C-3a), 123.9 (C-2), 121.0 (C-6), 118.5 (C-5), 118.3 (C-4), 111.4 (C-7), 108.6 (C-3), 46.1 (C-15^a^), 44.5 (C-13^a^), 35.7 (C-11), 32.7 (C-8), 26.2 (C-12), 22.7 (C-16); (+)-HRESIMS *m/z* 517.3277 [M + H]^+^ (calcd for C_30_H_41_N_6_O_2_, 517.3286).

#### 3.2.34. *N*^1^,*N*^8^-Bis(3-(2-(1*H*-indol-3-yl)acetamido)propyl)octane-1,8-diaminium 2,2,2-trifluoroacetate (**37**)

Using general procedure B, reaction of **34** (9 mg, 12 μmol) in CH2Cl2 (1.7 mL) with TFA (0.3 mL) followed by purification by LH20 column chromatography (MeOH) afforded **37** as a brown oil (6 mg, 90% yield).

R*_f_* = 0.46 (EtOAc); IR ν_max_ (ATR) 3277, 2940, 1672, 1132, 1023, 721 cm^−1^; ^1^H NMR (CD_3_OD, 400 MHz) δ_H_7.57 (1H, d, *J* = 8.0 Hz, H-4), 7.37 (1H, d, *J* = 8.2 Hz, H-7), 7.21 (1H, s, H-2), 7.12 (1H, ddd, *J* = 8.2, 8.2, 1.0 Hz, H-6), 7.03 (1H, ddd, *J* = 8.2, 8.0, 1.0 Hz, H-5), 3.69 (2H, s, H_2_-8), 3.31–3.27 (2H, m, H_2_-11), 2.78 (2H, t, *J* = 6.8 Hz, H_2_-13), 2.75–2.70 (2H, m, H_2_-15), 1.79 (2H, tt, *J* = 6.8, 6.8 Hz, H_2_-12), 1.60–1.50 (2H, m, H_2_-16), 1.43–1.27 (4H, m, H_2_-17 and H_2_-18); ^13^C NMR (CD_3_OD, 100 MHz) δ_C_ 176.5 (C-9), 138.2 (C-7a), 128.4 (C-3a), 125.2 (C-2), 122.7 (C-6), 120.1 (C-5), 119.3 (C-4), 112.6 (C-7), 109.4 (C-3), 48.8 (C-15), 46.0 (C-13), 36.7 (C-11), 34.0 (C-8), 29.9 (C-18), 27.6 (C-12), 27.3 (C-17^a^), 27.1 (C-16^a^); (+)-HRESIMS *m/z* 573.3899 [M + H]^+^ (calcd for C_34_H_49_N_6_O_2_, 573.3912).

#### 3.2.35. *N*^1^,*N*^12^-Bis(3-(2-(1*H*-indol-3-yl)acetamido)propyl)dodecane-1,12-diaminium 2,2,2-trifluoroacetate (**38**)

Using general procedure B, reaction of **35** (10 mg, 12 μmol) in CH_2_Cl_2_ (1.7 mL) with TFA (0.3 mL) followed by purification using LH20 column chromatography to afford **38** as a pink oil (8 mg, 92% yield).

R*_f_* = 0.20 (CH_2_Cl_2_:MeOH:TEA 4:1:0.01); IR ν_max_ (ATR) 3319, 2929, 1672, 1433, 1133, 721 cm^−1^; ^1^H NMR (CD_3_OD, 400 MHz) δ_H_ 7.59–7.56 (1H, m, H-4), 7.39–7.35 (1H, m, H-7), 7.21 (1H, s, H-2), 7.12 (1H, ddd, *J* = 8.3, 8.0, 1.2 Hz, H-6), 7.03 (1H, ddd, *J* = 8.3, 8.0, 1.0 Hz, H-5), 3.69 (2H, s, H_2_-8), 3.31–3.26 (2H, m, H_2_-11), 2.77 (2H, t, *J* = 7.1 Hz, H_2_-13), 2.74–2.68 (2H, m, H_2_-15), 1.78 (2H, tt, *J* = 7.1, 6.8 Hz, H_2_-12), 1.59–1.49 (2H, m, H_2_-16), 1.36–1.29 (8H, m, H_2_-17 to H_2_-20); ^13^C NMR (CD_3_OD, 100 MHz) δ_C_ 176.4 (C-9), 138.2 (C-7a), 128.4 (C-3a), 125.2 (C-2), 122.7 (C-6), 120.1 (C-5), 119.3 (C-4), 112.6 (C-7), 109.4 (C-3), 48.8 (C-15), 46.0 (C-13), 36.7 (C-11), 34.0 (C-8), 30.6 (C-18^a^), 30.5 (C-19^a^), 30.2 (C-20^a^), 27.6 (C-12), 27.5 (C-17^a^), 27.2 (C-16); (+)-HRESIMS *m/z* 629.4553 [M + H]^+^ (calcd for C_38_H_57_N_6_O_2_, 629.4538). 

### 3.3. Biological Assays

#### 3.3.1. *In Vitro* Anti-Protozoal Activity

The *in vitro* activities against the protozoan parasites *T.b. rhodesiense*, and *P. falciparum* and cytotoxicity assessment against L6 cells were determined as reported elsewhere [29]. The following strains, parasite forms and positive controls were used: *T.b. rhodesiense*, STIB900, trypomastigote forms, melarsoprol, IC_50_ of 0.005 μM; *P. falciparum*, NF54, erythrocytic stages, chloroquine, IC_50_ of 0.004 μM and L6 cells, rat skeletal myoblasts, podophyllotoxin, IC_50_ of 0.019 μM.

#### 3.3.2. *In Vivo* Anti-Malarial Efficacy Studies

*In vivo* anti-malarial activity was assessed as previously described [30]. Groups of three female NMRI mice (20–22 g) were intravenously infected with 2 × 10^7^ parasitized erythrocytes on day 0 with GFP-transfected *P. berghei* strain ANKA [32]. Compounds were formulated in 100% DMSO, diluted 10-fold in distilled water and administered intraperitoneally in a volume of 10 mL·kg^−1^ on four consecutive days (4, 24, 48 and 72 h post infection). Parasitemia was determined on day 4 post infection (24 h after last treatment) by FACS analysis. Activity was calculated as the difference between the mean per cent parasitaemia for the control (*n* = 5 mice) and treated groups expressed as a per cent relative to the control group. The survival of the animals was usually monitored up to 30 days: a compound was considered curative if the animal survived to day 30 after infection with no detectable parasites. *In vivo* efficacy studies in mice were conducted according to the rules and regulations for the protection of animal rights (“Tierschutzverordnung”) of the Swiss “Bundesamt für Veterinärwesen”. They were approved by the veterinary office of Canton Basel-Stadt, Switzerland.

## 4. Conclusions

The polyamine marine natural products didemnidine A (**2**) and B (**3**) have been previously identified as weak *in vitro* growth inhibitors of *Trypanosoma brucei rhodesiense* and *Plasmodium falciparum*. A series of 1, ω-substituted polyamine analogues were prepared that explored the influence of “capping acids” indole-3-glyoxylic acid and indole-3-acetic acid, length of polyamine chain and the presence or absence of mid-chain nitrogen substitution on antiprotozoal activity. Three analogues, one containing a PA3-8-3 core (**20**) and two containing PA3-12-3 cores (**29**, **32**) were identified as particularly potent antimalarials, with the former example also exhibiting good selectivity. Several analogues were identified that exhibit more enhanced anti-*Trypanosoma brucei* activity than the original natural product hits, but these same analogues also exhibited cytotoxicity, making them poorly selective. PA3-8-3 analogue **20** was only mildly active against *P. berghei* infection in a mouse model.

## Supplementary Files

Supplementary File 1 Supplementary Materials (ZIP, 26 KB)
